# Population Genetic Structure and Reproductive Strategy of the Introduced Grass *Centotheca lappacea* in Tropical Land-Use Systems in Sumatra

**DOI:** 10.1371/journal.pone.0147633

**Published:** 2016-01-25

**Authors:** Ladislav Hodac̆, Fuad Bahrul Ulum, Nicole Opfermann, Natalie Breidenbach, Diego Hojsgaard, Sri Sudarmiyati Tjitrosoedirdjo, Barbara Vornam, Reiner Finkeldey, Elvira Hörandl

**Affiliations:** 1 Department of Systematics, Biodiversity and Evolution of Plants (with Herbarium), Georg August University Göttingen, Göttingen, Germany; 2 Department of Biology, Faculty of Mathematics and Natural Sciences, IPB, Bogor Agricultural University, Bogor, Indonesia; 3 Department of Forest Genetics and Forest Tree Breeding, Georg August University Göttingen, Göttingen, Germany; University of Innsbruck, AUSTRIA

## Abstract

Intensive transformation of lowland rainforest into oil palm and rubber monocultures is the most common land-use practice in Sumatra (Indonesia), accompanied by invasion of weeds. In the Jambi province, *Centotheca lappacea* is one of the most abundant alien grass species in plantations and in jungle rubber (an extensively used agroforest), but largely missing in natural rainforests. Here, we investigated putative genetic differentiation and signatures for adaptation in the introduced area. We studied reproductive mode and ploidy level as putative factors for invasiveness of the species. We sampled 19 populations in oil palm and rubber monocultures and in jungle rubber in two regions (Bukit Duabelas and Harapan). Amplified fragment length polymorphisms (AFLP) revealed a high diversity of individual genotypes and only a weak differentiation among populations (*F*_*ST*_ = 0.173) and between the two regions (*F*_*ST*_ = 0.065). There was no significant genetic differentiation between the three land-use systems. The metapopulation of *C*. *lappacea* consists of five genetic partitions with high levels of admixture; all partitions appeared in both regions, but with different proportions. Within the Bukit Duabelas region we observed significant isolation-by-distance. Nine AFLP loci (5.3% of all loci) were under natural diversifying selection. All studied populations of *C*. *lappacea* were diploid, outcrossing and self-incompatible, without any hints of apomixis. The estimated residence time of c. 100 years coincides with the onset of rubber and oil palm planting in Sumatra. In the colonization process, the species is already in a phase of establishment, which may be enhanced by efficient selection acting on a highly diverse gene pool. In the land-use systems, seed dispersal might be enhanced by adhesive spikelets. At present, the abundance of established populations in intensively managed land-use systems might provide opportunities for rapid dispersal of *C*. *lappacea* across rural landscapes in Sumatra, while the invasion potential in rainforest ecosystems appears to be moderate as long as they remain undisturbed.

## Introduction

**Fig 1:**
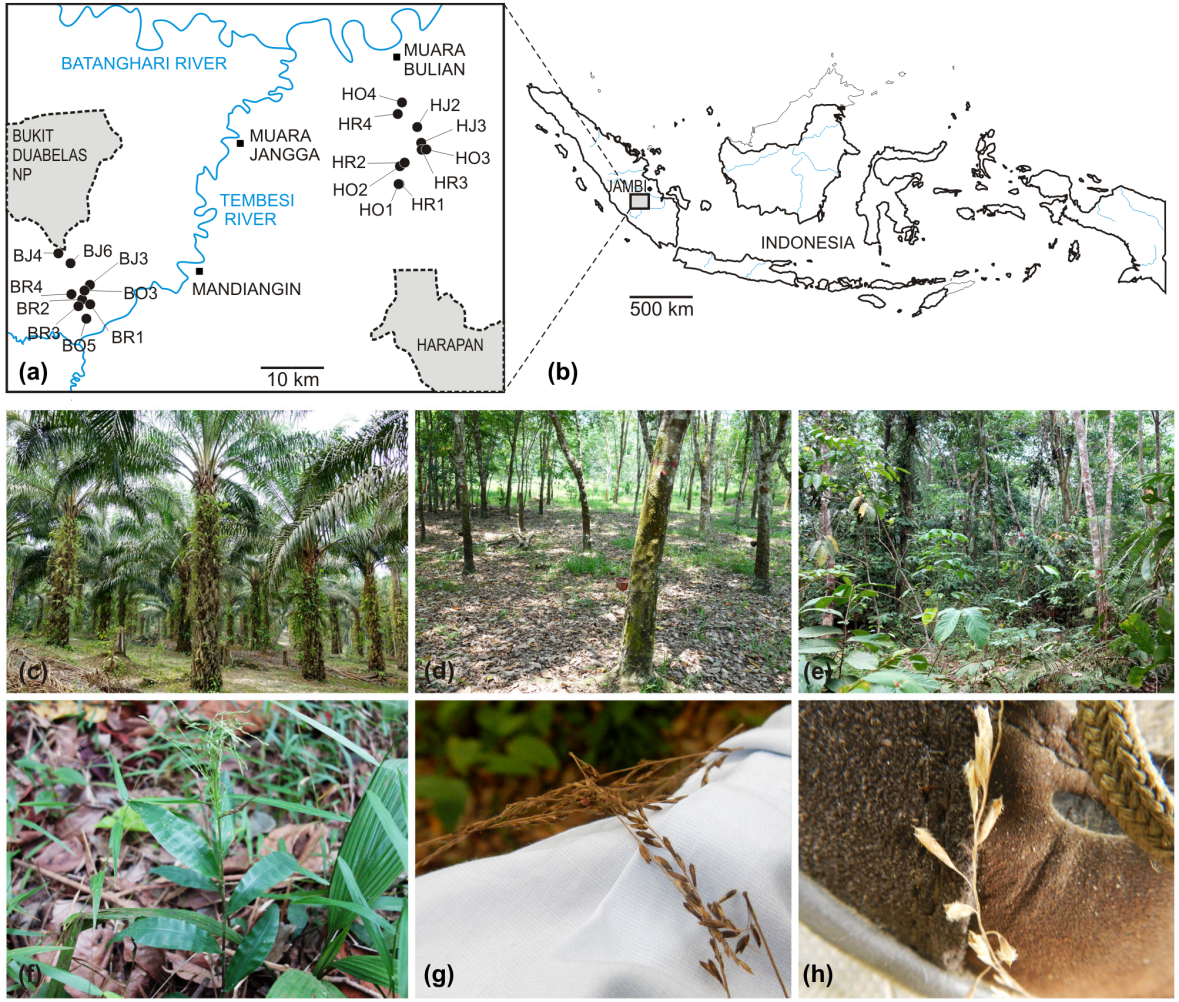
Sampling area and species under study, *Centotheca lappacea*. (a) An overview map showing nine sampling sites in Bukit Duabelas (BJ, BR, BO) and ten sampling sites in Harapan (HJ, HR, HO). The second letters in plot abbreviations correspond to three land-use systems (jungle rubber: “J”; rubber: “R”; oil palm: “O”). (b) Localization of the sampling area (Jambi province) in Sumatra Island (Indonesia). (c)-(e) Examples of sampling plots in the three land-use systems: (c) oil palm plantation, (d) rubber plantation and (e) jungle rubber system. (f) *Centotheca lappacea*, species under study. Effective epizoochory might be mediated by reflexed bristles on spikelets which easily lodge on clothes (g) and shoes (h) as photographed at Harapan sampling sites.

Tropical lowland rainforests shrink worldwide rapidly due to intensive ongoing deforestation [1]. In Sumatra (Indonesia), lowland rainforest was cut massively in the 1970s and 1980s and transformed into rubber and oil palm plantations, leaving only few remnants of natural forest, which are predominantly located in national parks [2] (Fig 1a and 1b). Beside monocultures of oil palm (*Elaeis guineensis*), and rubber (*Hevea brasiliensis*), an extensive management system of rubber trees planted into rainforest, also called “jungle rubber”, was established in the early 20^th^ century [2] (Fig 1c, 1d and 1e). The understory vegetation of such land-use systems is usually rapidly colonized by herbaceous weeds [3]. Alien species rapidly establish populations and may influence the native flora (“invasiveness” sensu [4]), but also native species can colonize novel anthropogeneous habitats in which they were not present before (“colonizers” sensu [4]). Displacement of native biota, change of ecosystems, environmental disturbance and hybridization with native species are the major threats of invasive plants to the maintenance of tropical ecosystems (e.g., [3]).

Invasion history is an important factor influencing population genetic structure and diversity [5]. Many studies compared genetic diversity between the native and the invasive range, but often failed to find differences between native and invasive areas (e.g., [6–9]). In the invaded area, species often show reduced genetic variation, which is in general referred to genetic bottlenecks after colonization because of founder effects and geographical isolation from source populations, especially after long distance dispersal [10]. However, multiple introductions can rapidly increase genetic diversity [7, 11, 12, 13]. After the initial introduction phase and a lag period of genetic adjustment, a phase of spread will rapidly expand the distribution range of the alien species [5]. Subsequently, isolation-by-distance mechanisms may initiate geographical differentiation within the invaded area.

Genetic diversity in the invaded area can enhance the adaptive potential to a novel environment [7]. Introduced plants will be exposed to novel stress situations, and will be under selection pressure on adaptive features. Indeed, niche shifts have been documented for many invasive plant species [14]. This adaptation process is also expected to become apparent only after a certain residence time and distribution over larger areas [5]. Standing genetic variation, or novel mutations enable plants to adapt to novel ecological niches and to establish in habitats of the invaded area [5]. Under this aspect, it is expected that populations differentiate genetically according to ecological conditions, because gene loci under selection would differentiate via beneficial mutations [15]. Adaptation may relate to local natural conditions of the invaded area (e.g., climate, soil and bedrock type, or geomorphological structure). On the other hand, the type of plantation and the management of land-use systems can change ecological conditions. For instance, carbon content of soil and degree of erosion differ between land-use systems in Sumatra [16]. Despite the fact that invasive species in land-use systems are widely distributed in the tropics, the information on genetic structure and adaptive potentials of herbaceous alien plants in tropical land-use systems remains insufficient [3].

Polyploidy is another important factor influencing genetic diversity and distribution of invasive plants. Many authors stress the positive effects of polyploidy for invasions (e.g., increased heterozygosity, vigor, life span, seed longevity, and seedling survival; [5]), and indeed polyploids are more frequent than diploids in the invasive plant floras of the temperate zones [17]. However, the information on ploidy levels of tropical invasive species is too leaky to make general conclusions.

Uniparental reproduction is another potential factor which makes both asexual and selfing plants pre-adapted to invasions [18–20]. Apomictic species and selfers can found populations via single individuals and are therefore potentially better colonizers than related outcrossers; this colonization ability is most efficient after long distance-dispersal of seeds (Baker’s law; [21]). Vegetative propagation, in contrast, remains in terrestrial biota usually spatially restricted, but is also an efficient local reproductive strategy of invasive plants [17]. Uniparental reproduction, however, would result in reduced genetic diversity or even clonality, and could reduce adaptive potentials to novel environments.

We investigate the model system *Centotheca lappacea* (L.) Desv. (subfamily Panicoideae, family Poaceae [22]), a perennial grass with 30–100 cm long erect culms (Fig 1f). Native to west tropical Africa, tropical to temperate Asia, Australia, and the Pacific islands [23, 24], it is widely used as a forage grass [25]. Although *Centotheca lappacea* was reported from natural rainforests of Thailand or Malaysia [26, 27], this species grows in Indonesia mainly as a weed in clearings, forest edges and paths, road sides, waste places, cocoa, oil palm and rubber plantations [28]. The species is the most frequent grass in the understory of oil palm and rubber plantations in the investigated regions of the Jambi province in Sumatra, but was observed only once in natural forests in the National Parks of the Jambi region (author’s team, pers. obs.). In contrast to other invasive grasses, the species colonizes not only intensively used monocultures, but also the more natural “jungle rubber” systems (S1 Table). Other than in Sumatra, *C*. *lappacea* was also reported from Kalimantan to dominate oil palm plantations, being used for testing herbicides [29] or from non-natural shrub vegetation in Vietnam [30]. Some authors regard the species in reserved areas as “invasive alien” [31]. However, the species is not yet recorded in the Global Invasive Species Database [32], and its actual invasive potential needs to be investigated.

The first documentation of the species in herbaria in Sumatra dates back to the mid of the 19^th^ century, but it was regularly collected only from the 1920s onwards (Global Biodiversity Information Facility, GBIF, as of December 2014 [33]). This time period coincides with the establishment of rubber (from 1904 onwards) and oil palm plantations in Sumatra (from 1913 onwards; [34]). Thus, after a residence time of c. 100 years, genetic bottlenecks probably have been overcome and first signatures of genetic differentiation and adaptation should be apparent in the introduced area. However, the factors for the wide distribution and abundance of this species were so far unknown, and no population genetic study is yet available for this species.

With respect to abundance of *C*. *lappacea* in intensively managed landscapes and disturbed habitats, the species might take advantage of selfing or an asexual reproductive mode, enhancing colonization abilities and invasiveness—a phenomenon well documented for other plant species [17]. Despite reports for a chromosome number 2n = 24 [35, 36], Levy (2002) suggested that the species might be polyploid [37]. Although many related widespread panicoid grasses reproduce asexually via apomixis [38], no study of mode of reproduction was so far available for *C*. *lappacea*, and the genus was not recorded in the Apomixis database (www.apomixis.uni-goettingen.de/) [39].

The aims of this study are 1) to analyze whether population genetic diversity and degree of divergence in the invaded range would fit to measures of established populations, 2) to test a hypothesis of geographical substructure of populations, 3) to identify candidate loci under natural selection as potential signatures of local adaptation to landscape heterogeneity and/or management types; 4) to assess ploidy level and reproductive strategies of *Centotheca lappacea* as putative factors enhancing invasiveness, and 5) to discuss the general invasive potential of the species.

## Material and Methods

### Ethic statement

The study was conducted using samples collected based on Collection Permit No. 3924/IT3/PL/2013 recommended by the Indonesian Institute of Sciences (LIPI) and issued by the Ministry of Forestry (PHKA).

### Plant material

The sampling sites were established in the frame of the project Collaborative Research Centre CRC990 “Ecological and Socioeconomic Functions of Tropical Lowland Rainforest Transformation Systems (Sumatra, Indonesia)”comprising four replicate sampling sites (50 x 50 m) for each land-use/transformation system (oil palm, rubber plantation, jungle rubber) and for natural forests in two landscapes of Jambi province, Bukit Duabelas and Harapan (http://www.uni-goettingen.de/en/310995.html; Fig 1a and 1b). In the framework of recording the complete understory vegetation by the CRC990 and investigating the ten most abundant invasive herbaceous species present in these plots, we identified *Centotheca lappacea* as the most abundant grass (according to presence/absence of the species). The species occurred in all 24 land-use plots (in five plots with less than five individuals), but only in one of eight natural forest plots. Leaves, flower bud fixations and seeds of *Centotheca lappacea* were collected during 2013 (May–September) from 19 spatially separated sites, treated here as distinct populations. Individual plants were sampled at a minimum distance of 5–10 m to each other to avoid the sampling of putative clones. We further had to restrict the sampling to flowering or fruiting plants for assessment of mode of reproduction. Our target was to sample at least 10 plants per plot, but the restrictions of a minimum-distance and of flowering/fruiting plants resulted in less than 10 samples in some of the plots. Altogether we collected a total of 173 individuals in 19 land-use plots (Table 1; Fig 1a and 1b).

**Table 1:** Mean values of population genetic parameters for *Centotheca lappacea*.

Population ID	Population size	Total bands	Private bands	Polymorphic bands	Haplotypes	PLP ^a^	H_j_ ^b^	H_s_ ^c^
BJ3	9	109	1	43	8	25.3	0.1222	0.1345
BJ4	8	124	8	60	8	72.9	0.1821	0.1565
BJ6	7	102	1	39	7	60.0	0.1418	0.1395
BO3	8	113	1	54	8	66.5	0.1739	0.1541
BO5	9	102	0	34	9	20.0	0.1111	0.1269
BR1	9	104	0	34	9	20.0	0.1118	0.1260
BR2	10	105	3	34	8	20.0	0.0876	0.1225
BR3	10	104	0	38	10	22.4	0.1134	0.1272
BR4	10	102	2	36	9	21.2	0.1119	0.1258
HJ2	10	106	0	35	9	20.6	0.1113	0.1328
HJ3	5	97	1	22	5	12.9	0.0987	0.1294
HO1	10	106	0	41	10	62.4	0.1353	0.1379
HO2	9	104	3	39	9	61.2	0.1319	0.1364
HO3	10	105	0	36	10	21.2	0.1083	0.1277
HO4	10	102	0	35	9	20.6	0.1140	0.1345
HR1	10	104	0	35	10	20.6	0.1056	0.1268
HR2	10	107	2	42	10	24.7	0.1252	0.1320
HR3	9	105	6	30	9	17.6	0.0929	0.1313
HR4	10	99	0	24	7	14.1	0.0600	0.1180
Mean	9	105	1	37	9	31.8	0.1178	0.1326

Abbreviation scheme for population IDs: B = Bukit Duabelas, H = Harapan; J = jungle rubber, O = oil palm monoculture, R = rubber monoculture.

^a^ PLP: proportion of polymorphic loci;

^b^ H_j_: expected heterozygosity (Nei's gene diversity, HWE assumed);

^c^ H_s_: panmictic heterozygosity (no HWE assumed).

### DNA extraction and AFLP fingerprinting

Genomic DNA was extracted from (silica gel dried) leaf material using the DNeasy 96 Plant kit (Qiagen, Hilden, Germany) following manufacturer´s instructions. The total of 173 samples were analyzed using standard AFLP protocols [40] with a slight modification and two selective AFLP primer combinations. The restriction enzymes EcoRI and MseI were incubated with 4μl DNA solution simultaneously overnight. For preselective amplification reaction the primers E01/M03 (A-3’/G-3’) were used and 4μl restriction-ligation solution was added to the PCR reaction. The PCR product was diluted in water and processed with two combinations of selective primers: E35/M63, E32/M66. EcoRI/MseI primer combination E35/M63 contains the three nucleotides ACA/GAA as the specific extension and E32/M66 the nucleotides AAC/GAT at the 3’end of the AFLP primer. Prior to fragment analysis in Genetic Analyzer 3130, ABI PRISM, the PCR product was diluted in water and 12μl HiDi formamide. As a size standard GENSCAN 500 ROX was added to the solution, while the fragments were labeled with the fluorescent FAM marker. Fragment scoring was carried out in the program GeneMarkerV2.4.2 (© SoftGenetics, LLC) and fragments of 50–500 bp in length were scored. AFLP multi-locus genotypes were scored using '0' for the absence and '1' for the presence of a locus. Reproducibility of AFLP profiles was assessed by repeating independent DNA extractions and AFLP amplifications [41, 42]. We repeated 50% of randomly chosen individuals. Only fragments which could be unambiguously recognized as present or absent in the replicated individuals were scored by applying a scoring threshold of 10% of the highest peak’s intensity within the locus under consideration [43]. Samples with poor DNA quality were not considered for the analysis. The final binary matrix comprised 173 individuals from 19 populations scored at 170 loci.

### Estimation of genetic diversity

Genetic diversity at population level was estimated as follows: 1) number of private bands and number of polymorphic loci per population were calculated using FAMD 1.31 [44], 2) amount of AFLP haplotypes per population was counted using Arlequin 3.5 [45], 3) proportion of polymorphic loci (PLP) at the 5%-level and Nei's gene diversity (H_j_; assuming Hardy—Weinberg equilibrium/HWE) were computed with AFLP-SURV 1.0 [46] and 4) average panmictic heterozygosity (H_s_; no HWE assumption) was computed with Hickory 1.1 [47]. Measures of genetic diversity across populations were compared between Bukit Duabelas and Harapan employing a non-parametric t-test (1000 permutations) in the software PAST 2.17c [48]. Similarity among individual genotypes was visualized via Neighbor-joining tree (incl. bootstrap support values inferred from 1000 replicates) based on standard Simple matching coefficient (SMC) using FAMD 1.31 [44]. We compared the overall genetic diversity within each land-use system based on Nei's gene diversity (H_j_; S1a Fig) and panmictic heterozygosity (H_s_; S1b Fig). Differences of land-use median values were tested by a Kruskal-Wallis test and Mann-Whitney pairwise comparisons (uncorrected and Bonferroni corrected *P*-values) in PAST 2.17c [48]. In the same program, we tested the diversity indices for correlation using Spearman´s ρ coefficient. To check, whether unequal population sizes inflate allelic diversity estimates, we used AFLPdiv [49] and computed band richness within each population after rarefaction to 5 (the smallest population size; S2 Fig).

### Genetic differentiation and loci under selection

To analyze genetic differentiation, genetic variation was partitioned and tested by Analysis of Molecular Variance (AMOVA) using Arlequin 3.5 [45]. First, total genetic diversity was partitioned among populations (*F*_*ST*_) between Bukit Duabelas and Harapan regions. Second, we conducted another AMOVA to infer differentiation among (*F*_*CT*_) and within (*F*_*SC*_) land-use systems. To detect candidate loci under natural selection departing from a neutral model [15, 50, 51], we analyzed the dataset of 170 loci by using BayeScan 2.1 [50] and parameters set as default. Nine loci with a threshold probability ≥ 0.99 of being under diversifying selection (decisive evidence, [50]) were identified by the BayeScan algorithm. To get insights into their contribution to geographical differentiation, we analyzed these loci by Principal component analysis (PCA) using the program PAST 2.17c [48]. In order to support results from BayeScan, the divergence outlier analysis (DOA) was conducted with the program Mcheza [52]. Genetic structure of populations from Bukit Duabelas and Harapan was further analyzed using STRUCTURE 2.3.4. The Bayesian analysis based on all 170 loci was performed applying an admixture model, a burn-in of 500,000 generations and a subsequent run length of 700,000 generations, testing values of K (assumed number of genetic populations) between 1 and 10 with 20 replicates per K value. To evaluate the fit of different clustering scenarios (best K) we analyzed the mean log probability, L(K) [53], and the change in the log probability, ΔK [54]. Alternatively to the Bayesian inference, a method for quick mapping of admixture without source samples was employed in the clustering program FLOCK [55]. In contrast to Structure, instead of employing the MCMC algorithm, FLOCK uses an iterative method based on allele frequencies [56]. The datasets from Bukit Duabelas and Harapan were each tested for isolation-by-distance (IBD) by correlating pairwise *F*_*ST*_ values (Arlequin 3.5 [45]) with geographic distances (in km) employing a Mantel test in PASSaGE [57]; the significance was tested after 1000 permutations. Geographic distances inferred from GPS coordinates (S1 Table) were computed in Geographic Distance Matrix Generator v1.2.3 [58].

### Ploidy level

To assess the chromosome number on a representative subset of plants, a set of ten seeds per three different land-use systems were germinated in soil inside climate growth chambers at 25°C. Three root tips in active growth from each of eight cultivated plants were pretreated with a saturated aqueous solution of α-bromonaphthalene for three hours at room temperature. Selected root tips were fixed for 12–24 hours in three absolute ethanol: one glacial acetic acid and then conserved in 70% ethanol at 4°C. Most of the pre-treated materials were directly hydrolyzed with 1N HCl at 60°C for 10 min and stained with basic fuchsine. Feulgen staining following methods by [59] was performed for chromosome counting. Meristem cells were macerated in a drop of 2% aceto-orcein and then squashed. Cells in mitotic stages were observed under a Leica DM 5500B Microscope in 1000x magnification (Leica Microsystems GmbH, Wetzlar, Germany), the total chromosomes in a cell were counted to define the number of chromosomes and the ploidy level. Representative images were taken with a DFC450C camera (Leica Microsystems GmbH, Wetzlar, Germany).

To assess the ploidy level of the whole sampling, flow cytometry was performed on 164 plants by using 0.5 cm^2^ of dry leaves from field collections. As a reference we used fresh leaf tissue from a plant for which previously chromosomes were counted. A single leaf was chopped with a razor blade in a petri dish containing 200 μL Nuclei extraction buffer (Otto 1; [60]). The resulting suspension was filtered through 30-μm mesh Cell Tric disposable filter (Sysmex Partec GmbH, Münster, Germany), and stained with 800 μL staining buffer (Otto 2; [60]). On the next step of analysis we pooled two leaves from different individuals to make the analysis faster. Samples were analyzed using a Partec PA II Flow Cytometer (Sysmex Partec GmbH, Münster, Germany) with the UV-detector operating at 355 nm. Ploidy levels of the leaf tissues were estimated by comparing the sample peak to the standard peak. Approximately 3000 nuclei were measured per sample. Data analysis was performed using PA II’s Partec FloMax software. The mean values of DNA content of the leaves were established to infer the ploidy level of the sample. The coefficient of variation for each sampled peak was around 5% or less. A regression analysis for histogram data of dried leaves was applied to confirm that the mean values of G1 and G2 peaks had a linear correlation (*P* < 0.05). All statistical analyses were carried out with Statistica software version 10.; StatSoft, Inc. (2011).

### Cyto-embryological analysis

To test for sexual versus apomictic embryo sac development, a total of 338 spikelets from 16 different individuals was analyzed by microscopic observation of megasporogenesis and embryo sac development following methods described in Young et al. [61]. Inflorescences that were previously fixed in FAA and stored in ethanol 70% were dehydrated in 100% ethanol for 30 minutes. Afterwards, they were incubated in 300 μl of upgrading series of methyl salicylate (Merck KGaA, Darmstadt, Germany) diluted in ethanol (25%, 50%, 70%, 85%, and 100%) for 30 minutes in each steps. Spikelets were dissected to prepare the ovules and anthers, then ovules and anthers were amounted in methyl salicylate on glass slides. The stages of ovule and anther development were analyzed by using a Leica DM5500B microscope with Nomarski DIC optics in 400x magnification (Leica Microsystems GmbH, Wetzlar, Germany). Images were taken by a DFC450C camera (Leica Microsystems GmbH, Wetzlar, Germany).

### Flow cytometry seed analysis

To reconstruct sexual vs. apomictic pathways of seed formation, Flow Cytometric Seed Screen (FCSS) protocols described by [62] and [38] were used to analyze 310 mature seeds. A single seed from each individual was chopped with a razor blade in a petri dish containing 200 μL Nuclei extraction buffer (kit Cystain UV precise P, Sysmex Partec). The resulting suspension was filtered through 30-μm mesh Cell Tric disposable filter (Sysmex Partec GmbH, Münster, Germany), and stained with 800 μL staining buffer (kit Cystain UV precise P, Sysmex Partec). All samples were incubated for 30 sec to 60 sec on ice before measurement. In the next step of analysis we pooled five seeds from the same individual and measured DNA content as described above for leaves. The mean values of DNA content for embryo and endosperm of each measurement were calculated to infer the ploidy levels of embryo to endosperm. The coefficient of variation for each sampled peak was around 5% or less. The rationale for using FCSS is based on the different ratios of embryo to endosperm relative DNA content within seeds as a consequence of fertilization of unreduced (apomictic) versus reduced (sexual) embryo sacs [62]. In the sexual seed, double fertilization leads to the formation of a 2n (2C) embryo and a 3n (3C) endosperm (2: 3 ratio). In the apomictic pathway, fertilization occurs either only in the central cell of the unreduced embryo sac and produces a 2n (2C) parthenogenetic embryo and 5n (5C) or 6n (6C) pseudogamous endosperm (1n or 2n from sperm plus 4n from central cell; 2: 5 or 1: 3 ratio). Alternatively, apomixis can also be fertilization-independent (2n embryo and 4n endosperm; 1: 2 ratio) [62]. For flow cytometry data, the mean, minimum and maximum of the histogram peak values, and Pearson product-moment correlation for the mean values were calculated.

### Self-compatibility tests

Pollen-pistil self-compatibility was tested by bagging *C*. *lappacea* inflorescences of genotypes from different collection sites. Selected inflorescences were trapped in sulphite paper bags 2–5 days before flowering and harvested between 45 and 60 days after anthesis. Presence of full caryopses was checked with a Leica S6E stereoscope (Leica Microsystems GmbH, Wetzlar, Germany).

## Results

### AFLP data

#### Genetic diversity and AMOVA

**Fig 2:**
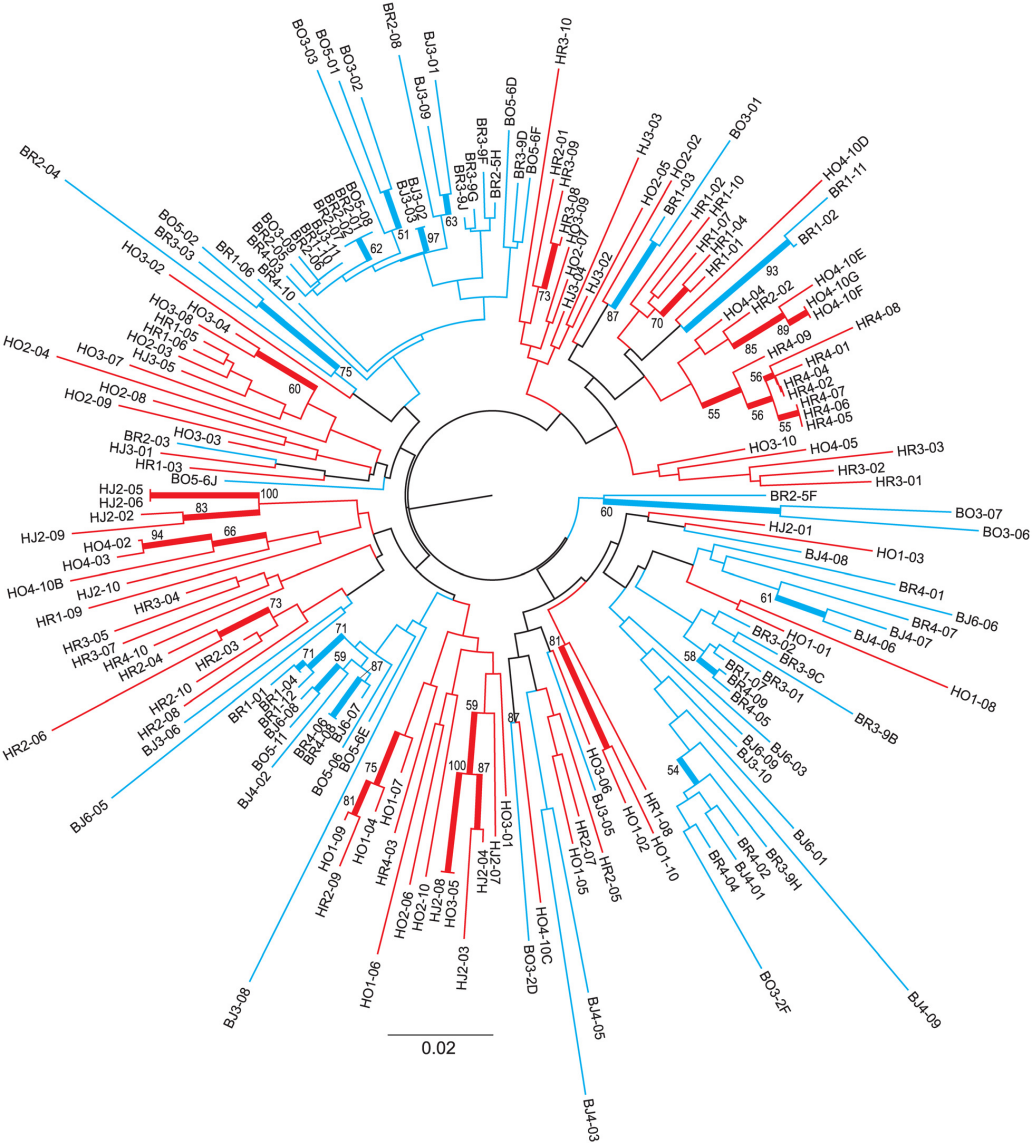
Neighbor-joining tree. Neighbor-joining tree based on standard SMC (Simple Matching Coefficient) similarities calculated from 170 AFLP loci representing 19 populations of *Centotheca lappacea*. Branches are colored according to sampling regions (blue: Bukit Duabelas, BJ/BR/BO individuals; red: Harapan, HJ/HR/HO individuals). Thick lines indicate branches with ≥ 50% bootstrap support.

**Fig 3:**
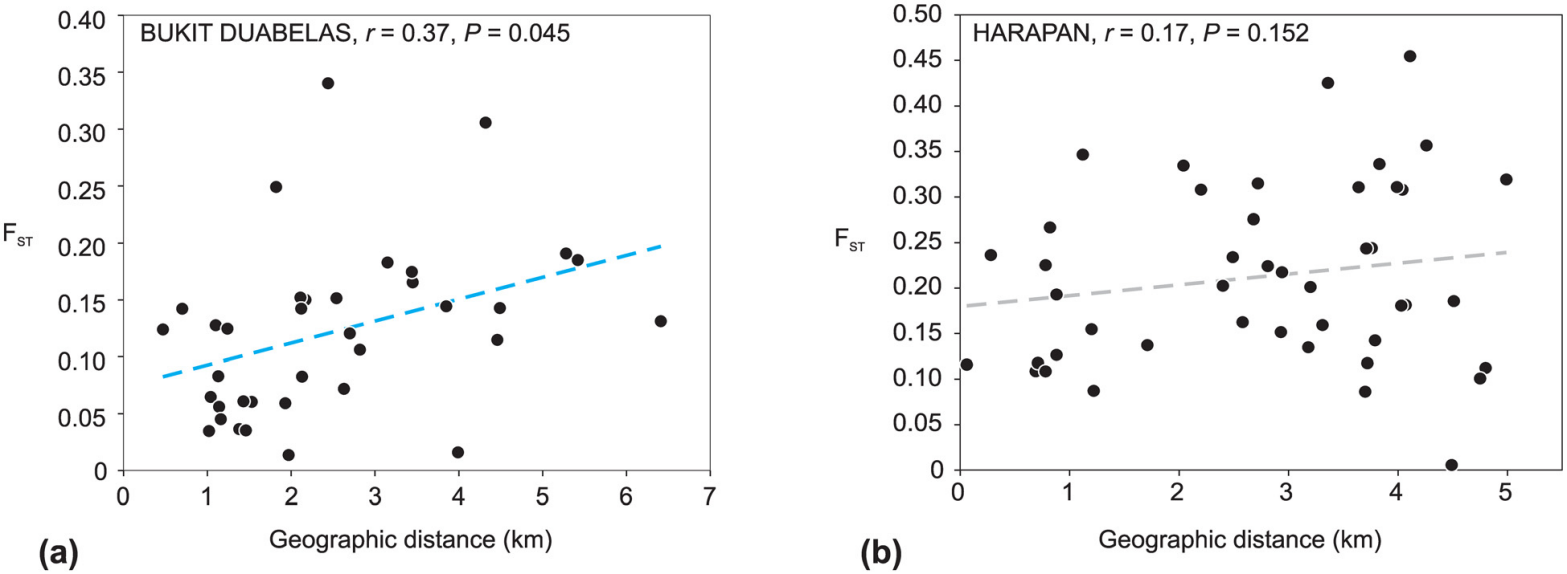
Analysis of isolation-by-distance. Isolation-by-distance (IBD) of *Centotheca lappacea* populations within Bukit Duabelas (a) and Harapan (b). Results of Mantel tests (two tailed *P*; 1000 permutations) are given. Black circles indicate pairs of populations and the blue dashed regression line indicates a significant correlation of genetic divergence and geographical distance.

In total, 173 individuals of *Centotheca lappacea* from 19 populations were scored and analyzed (Table 1; S1 Table). Two AFLP primer combinations resulted in 170 clearly scorable and reproducible fragments sized from 53–443 bp, and 74.71% of them were polymorphic. Measures of population genetic diversity (i.e., PLP, H_j_, H_s_; see values in Table 1) were intercorrelated (S2 Table) and revealed no significant difference between Bukit Duabelas and Harapan (PLP: t = 0.941, *P* = 0.317; H_j_: t = 1.634, *P* = 0.128; H_s_: t = 0.923, *P* = 0.370). However, rubber land-use systems differed significantly from both jungle rubber and oil palm by lower means of panmictic heterozygosity (H_s_; S1 Fig). Considering all 19 populations, genetic diversity was mostly distributed within populations rather than among populations (overall *F*_*ST*_ = 0.173, Table 2). The genetic variation was partitioned to 6.53% between Bukit Duabelas and Harapan and to 16.16% among populations within both regions (AMOVA; Table 2). Accordingly, the individuals did not cluster separately with respect to regions in the Neighbor-joining tree (Fig 2). These analyses further separated individual genotypes without any indication of clonality. Populations within land-use systems were moderately differentiated in both Bukit Duabelas (11.72%) and Harapan (19.70%) (AMOVA; Table 2), but no significant differentiation among land-use systems was found within regions. For Bukit Duabelas, we obtained a weak but significant positive correlation (*r* = 0.37, *P* = 0.045) between geographic distances and genetic divergence among populations (Fig 3a). In Harapan, no significant isolation-by-distance was observed (Fig 3b).

**Table 2:** Summary of Analysis of Molecular Variance (AMOVA) according to landscapes (Harapan; Bukit Duabelas) and land-use systems (jungle rubber, rubber and oil palm plantations).

	Overall					Bukit Duabelas					Harapan				
Source of variation	d.f.	% var.	*P*_(var.)_	F	*P*_(F)_	d.f.	% var.	*P*_(var.)_	F	*P*_(F)_	d.f.	% var.	*P*_(var.)_	F	*P*_(F)_
Among landscapes	1	6.53	***	0.065	***	-	-	-	-	-	-	-	-	-	-
Among populations within landscapes	17	16.16	***	0.173	***	2	5.04	***	0.050	***	2	7.90	***	0.079	***
Within populations (within landscapes)	154	77.31	***	-	-	77	94.96	-	-	-	90	92.10	-	-	-
Among land-use systems (LUS)	2	1.10	0.186	0.011	0.186	2	1.31	0.298	0.013	0.298	2	2.17	0.167	0.022	0.167
Among populations within LUS	-	-	-	-	-	6	11.72	***	0.119	***	7	19.70	***	0.201	***
Within populations (within LUS)	-	-	-	-	-	71	86.97	-	-	-	83	78.13	-	-	-

Abbreviations: d.f. = degrees of freedom; F = fixation index; % var = percentage of variation;

* = *P* < 0.05,

** = *P* < 0.01,

*** = *P* < 0.001.

#### Loci under selection and genetic structure

**Fig 4:**
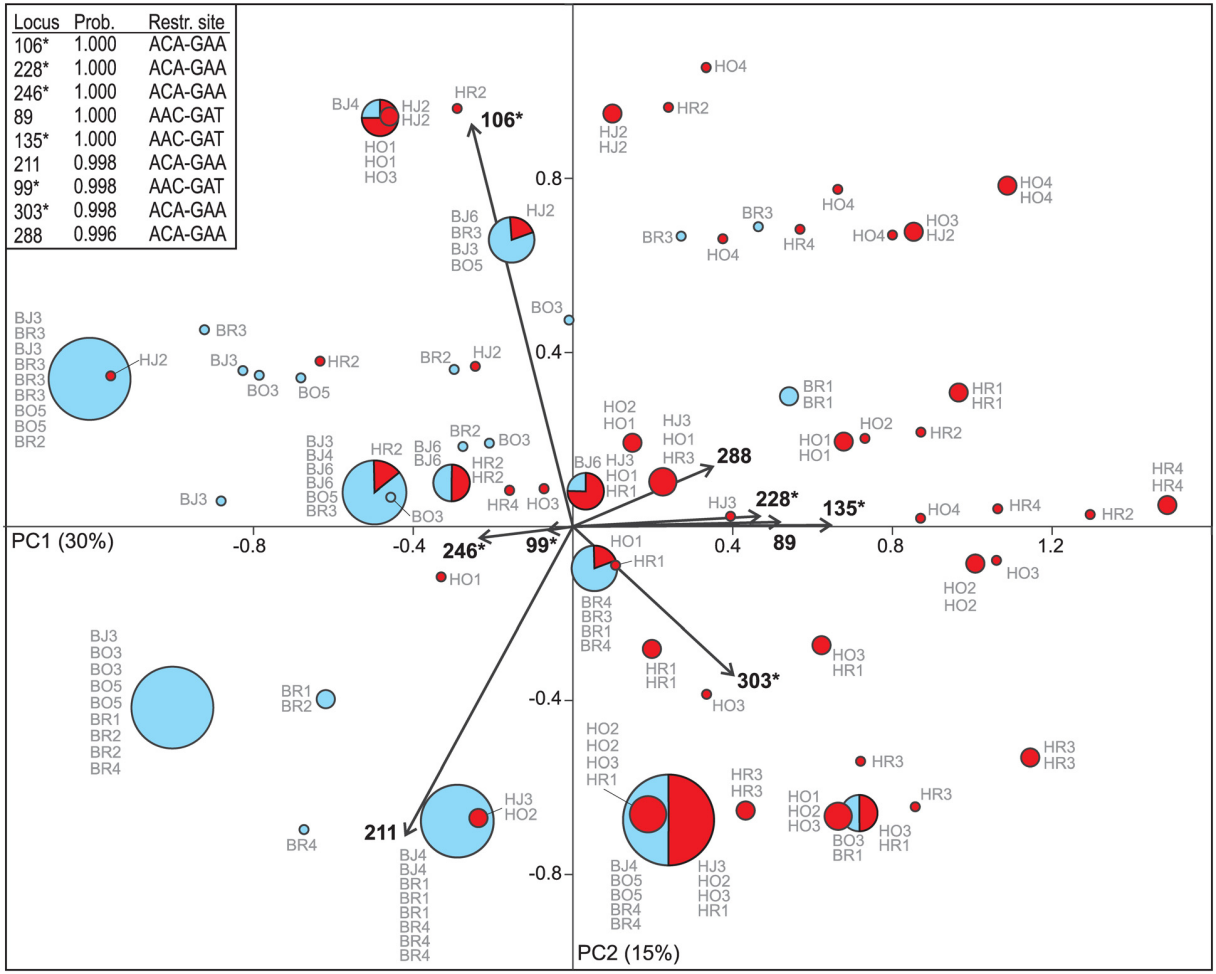
Principal component analysis (PCA) of nine loci under natural selection. Ordination of all 173 individuals based on nine AFLP loci detected by the program BayeScan. Each circle represents a single genotype and its size corresponds to the number of identical genotypes. The circles are colored according to two sampling regions (blue: Bukit Duabelas, BJ/BR/BO individuals; red: Harapan, HJ/HR/HO individuals). The nine loci exceeding a threshold for decisive evidence for selection (with probability “*P*” ≥ 0.99) are listed including their respective restriction sites (RS); loci concordantly depicted by the program Mcheza are marked by an asterisk.

**Fig 5:**
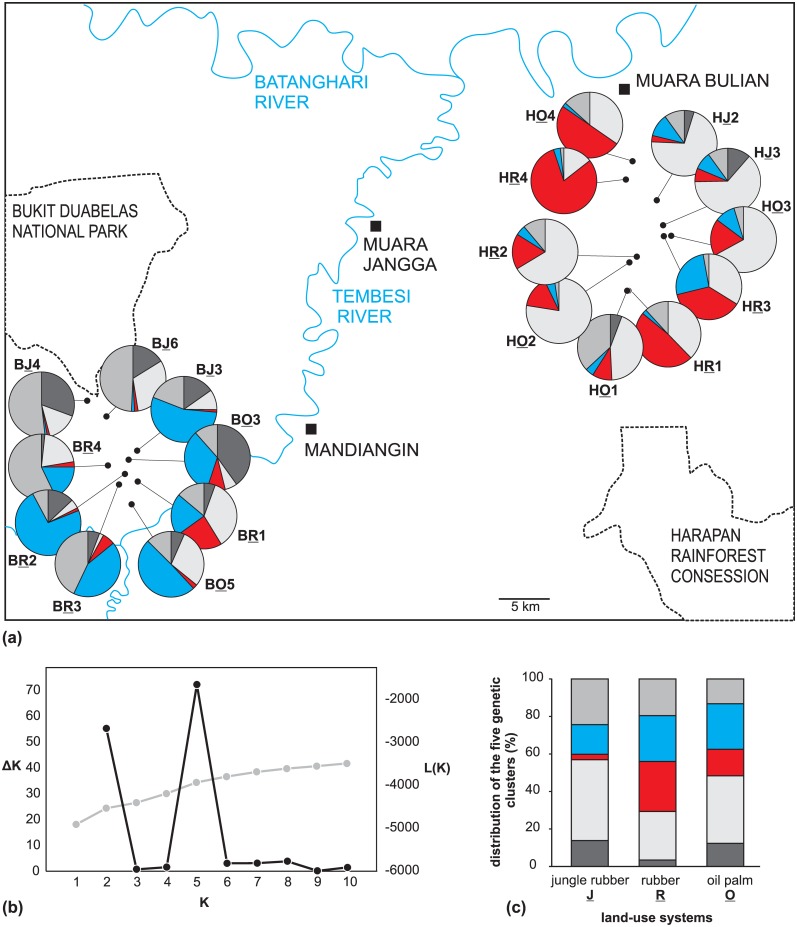
STRUCTURE analysis. Bayesian inference of genetic structure of the AFLP dataset comprising 19 populations from Bukit Duabelas (BJ/BR/BO) and Harapan (HJ/HR/HO); underlined letters indicate the land-use systems (jungle rubber = J, rubber = R, oil palm = O). (a) Genetic structure of populations based on five partitions (K = 5) inferred by STRUCTURE and visualized as pie charts. Red and blue colored partitions suggest modest divergence between Bukit Duabelas and Harapan. (b) Evanno plots of K’s (number of genetic clusters) against corresponding L(K) (grey dots) and ΔK (black dots). (c) Bar chart summarizing proportions of the five genetic partitions (colored as above) across the land-use systems.

Analysis in BayeScan identified nine non-neutral loci exceeding the threshold for decisive evidence of selection (posterior probability ≥ 0.99), all of them with a diversifying effect. Six out of these nine loci were concordantly depicted by an alternative search algorithm implemented in Mcheza. A principal component analysis (PCA) based on all nine loci from BayeScan shows a tendency of a separation between Bukit Duabelas and Harapan individuals along the first ordination axis (PC1, 30% of variation; Fig 4). In particular, the loci '228', '246', '135', '99', depicted by both programs, separate between the both regions. The model-based clustering (Structure analysis) recognized five distinct genetic partitions (best K = 5) with pronounced admixture (Fig 5a). According to both direct log-likelihood posterior probabilities and ΔK (Fig 5b), this assessment was unambiguous and 34.12% of individuals were assigned to a particular partition with a posterior probability ≥ 0.90, supporting strong admixture between the partitions. The absolute number of genetic partitions depicted by Structure was in congruence with an independent estimate given by non-Bayesian method implemented in FLOCK, suggesting the best K ≥ 4, and rejecting the second-best result for ΔK (K = 2) in the STRUCTURE analysis. Four of the five genetic partitions occurred in all 19 populations and differed only in their proportions between Bukit Duabelas and Harapan (Fig 5a). One partition occurred in all except for seven Harapan populations (Fig 5a, dark grey color). The five genetic partitions were present in all three land-use systems, exhibiting only moderate proportional differences (most pronounced in partition “3”, which is most frequent in rubber and less frequent in jungle rubber; Fig 5c, red color).

### Ploidy level and breeding system of *Centotheca lappacea*

**Fig 6:**
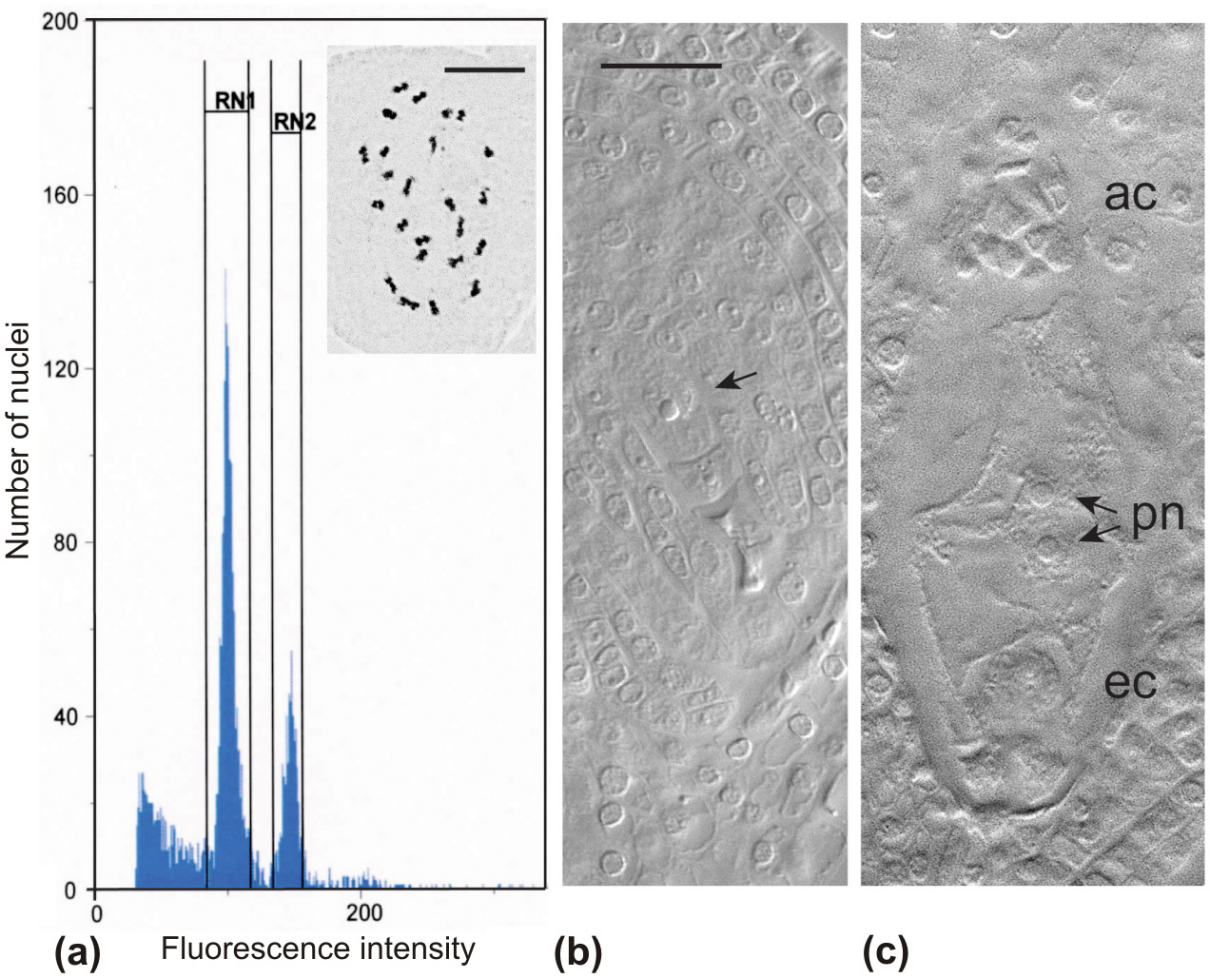
Ovule and seed development in *Centotheca lappacea*. (a) Single seed flow cytometry histogram of 3000 nuclei from genotype HJ3-01 showing the characteristic 2: 3 ratio (relative peak positions of ~100 and ~150 on the *x*-axis) between the embryo (RN1, diploid) and endosperm (RN2, triploid) ploidies corresponding to a seed formed via a sexual reproductive pathway. The mitotic metaphase cell showing 2*n* = 2*x* = 24 chromosomes corresponds to a diploid maternal plant from HJ3 population, and further supports diploidy on seed embryos. (b) End of megasporogenesis showing four meiotic products, the two toward the micropyle aborted. The arrows indicate the functional megaspore (from genotype BJ2-16). (c) Mature female gametophyte at the time of anthesis (from genotype BO1-2). After development of the characteristic *Polygonum*-type embryo sac carrying three antipodal cells, extra mitotic divisions produce a mass of antipodal cells (ac) at the chalazal embryo sac pole. Further abbreviations: ec (egg cell), pn (polar nuclei). Scale bars represent 20 μm.

The chromosome number of *C*. *lappacea* was 2n = 2x = 24 in all samples investigated (Fig 6a). Ploidy levels of *C*. *lappacea* were screened by flow cytometry of leaf material from field collections (S3 Table) using fresh leaf material of a plant with known chromosome number as standard (S4 Table). The fresh leaves of 21 diploid samples had a mean value of the first peak fluorescence intensity (G1) 100.37 ± 8.93, with a coefficient of variation (CV%) value less than 5%. DNA content of dried field-collected leaves of a total of 93 samples representing 123 individuals indicated the same ploidy level compared to those of the standard fresh leaves, with a mean value of the G1 peak of 92.01±7.24 (a slightly lower DNA content measurement in silica-gel dried leaves compared to fresh materials is a regular observation in flow cytometric studies and is due to DNA degradation (e.g., [63]). All samples were confirmed as diploid. There was no indication of polyploidy.

Microscopic analysis of ovules indicated obligate sexual reproduction without any evidence for apomixis. *Centotheca lappacea* develops after meiosis a tetrad of megaspores, but only the chalazal (functional) megaspore (Fig 6b and 6c) develops into a 7 celled and 8-nucleated *Polygonum*-type embryo sac (not shown; [64]). No aposporous initials were observed in the ovules. Formation of full caryopses was only observed in open pollinated inflorescences, while in bagging experiments only empty spikelets were recovered from a total of nine inflorescences (and around 5,200 spikelets) from six genotypes. Flow cytometric analysis on the seeds revealed consistently a 2: 3 ratio of embryo to endosperm ploidy (Fig 6a; S5 Table), suggesting that they originated sexually from double fertilization of an haploid egg cell and two reduced polar nuclei by haploid sperm nuclei. Altogether the data indicate that *C*. *lappacea* is a sexually reproducing species, and sexual seeds are formed via a *Polygonum*-type ovule development and allogamy due to the presence of a pollen-stigma incompatibility system.

## Discussion

We provide the first study on putative factors linked with invasiveness of the tropical grass *Centotheca lappacea*. Here, we focused on population genetic diversity, genetic structure, mode of reproduction, and ploidy level in three different agroforest systems in Sumatra.

Our population genetic study suggests that genetic diversity is in the invasive grass *C*. *lappacea* in Sumatra mostly distributed within populations (c. 83% of total variation). The *F*_*ST*_ value (0.173), estimating genetic differentiation among populations, is lower than grand means of plant populations in early successional stages (0.37) and more similar to late successional stages (0.23; values for dominant markers [65]). A low genetic differentiation (*F*_*ST*_ = 0.182, AFLP data) was also reported for sexual *Solidago canadensis* populations in the invasive range in China, where the species was introduced c. 85 years ago [66]. A scenario of post-colonization selection for a single genotype, as reported for the invasive asexual plant *Chromolaena odorata* [67], cannot be inferred from our data. Since we do not have any information on genetic diversity from the native areas of *C*. *lappacea*, we cannot reconstruct colonization history with our data alone. However, the distribution of diversity within populations would fit better to a scenario of established outcrossing populations rather than to recently founded populations [5, 11]. Herbarium collections indicate that the species appeared in Indonesia in the 2^nd^ half of the 19^th^ century and was regularly sampled from the 1910s and 1920s onwards [33], which coincides with the onset of jungle rubber and oil palm land-use systems in Sumatra). Accordingly, the species may have already overcome initial genetic bottlenecks and is at present in an advanced phase of establishment and adaptation to environmental heterogeneity in the introduced area.

Individuals do neither cluster in populations nor in geographical regions (Fig 2), and strong admixture exists between the genetic partitions that occur in both regions. This lack of geographical structure resembles the situation in other genetically diverse invasive weeds [13, 68]. Population genetic structure of *C*. *lappacea* is in the whole area partitioned into five gene pools, which all occur in both landscapes (Harapan and Bukit Duabelas), but with unequal proportions within the areas. The populations in Harapan were more homogeneous and showed no isolation-by-distance, while in Bukit Duabelas the populations showed a weak but significant geographical substructure, which may relate to a higher landscape heterogeneity in the hillside region of Bukit Duabelas. Altogether, the populations in the Jambi region appear still like a big “metapopulation” with only a weak geographical substructuring and continuous gene flow over the whole area. Interestingly, populations from rubber plantations in both landscapes exhibited a significantly lower gene diversity / heterozygosity compared to jungle rubber and oil palm (S1b Fig). Otherwise there was no indication of a genetic differentiation according to land-use systems.

We identified nine non-neutral AFLP loci under diversifying selection as identified by BayeScan, which represent 5.3% of all loci investigated here. This result resembles findings on a comparable AFLP dataset of the invasive species *Mikania micrantha*, in which 2.9% of loci were identified as outliers and interpreted as signatures for local adaptation [68]. Our study may have revealed candidate loci for a potential adaptation of *C*. *lappacea* after the time of spread, as predicted by theory [5]. The outlier loci showed a tendency to differentiate populations according to the two landscapes in the Principal Component Analysis (Fig 4). We hypothesize that candidate loci under selection in *Centotheca lappacea* may reflect the first fine-tuned signatures of regional adaptation to different ecological conditions in the two landscapes. Bukit Duabelas represents a more heterogeneous, hillside landscape, while Harapan represents a rather uniform plain landscape, which may have effects on abiotic ecological conditions within the plots. Moreover, soils in Bukit Duabelas are dominated by clay acrisol, while in Harapan they are dominated by sandy loam [16]. Hence, results of our study may provide a starting point for more detailed genetic studies on local adaptation.

The predominant distribution of genetic diversity within populations fits to an obligate sexual, outcrossing breeding system [65]. Sexual ovule development was observed by microscopic analysis, and obligate sexual seed formation was confirmed by flow cytometric seed screening. Bagging of inflorescences revealed no seed set and hence confirmed self-incompatibility. These results are surprising as other invasive Panicoid grasses in Sumatra were reported to be apomictic (e.g., *Paspalum conjugatum* [69, 70] and *Pennisetum polystachion* [71, 72]). According to field collections of the author’s team and individual genotypes observed in our AFLP data (Fig 2), also vegetative propagation of *Centotheca lappacea* can be ruled out as a factor for invasiveness. Since grasses are wind-pollinated, *C*. *lappacea* had obviously no pollinator limitation in the invaded area, and successfully established populations via outcrossing. Sexual reproduction may have rapidly restored genetic diversity after initial colonization phases with recurrent gene flow within and among populations, and natural selection can act efficiently on a diverse gene pool. Polyploidy and related genomic features, in contrast, can be ruled out as a factor for the invasion success of the species. Whether polyploidy is in tropical areas less relevant for invasion success than in temperate regions (e.g., [17]), needs to be studied.

Gene flow among populations of the species in land-use systems may have been enhanced by efficient seed dispersal mechanisms via spikelets with adherent bristles (Fig 1e and 1f). Diaspores adhering on clothing of farm-workers can easily be carried over longer distances by the use of cars or motorbikes in the area of plantations. The importance of human-mediated transportation of diaspores over roads, and its effects on population genetic structure, was also recognized in the invasive plant *Flaveria bidentis* [13]. Also a good germinability of seeds was observed in our cultivation experiments. These features may strongly contribute to the distribution success of a plant species [73].

Our study provides for the first time data on occurrence, population genetic structure, and mode of reproduction of *C*. *lappacea* in land-use systems in Sumatra. These basic data can be useful for estimating the potential of the species to invade natural rainforests. The species does occur in jungle rubber, it is also elsewhere shade-tolerant (e.g., [74]) and thus probably pre-adapted to the colonization of natural rainforest. Shade tolerance is an important factor for invasiveness, as light is in tropical forest understory the most limiting resource for plant growth [74]. From a population genetic view, *C*. *lappacea* successfully has established populations in agroforest systems, and probably can grow in natural forests as well. However, in the Jambi region the species was observed in only one of the eight investigated natural rainforest plots of National Parks. Within natural forest ecosystems, the scarcity of human visits, and the lack of human dispersal routes along roads and footpaths are probably major limiting factors for spread. As an outcrosser, the species cannot establish populations with a single erratic diaspore which might occasionally be dropped in the understory. In so far, the present potential of the species to invade natural, undisturbed forests may remain limited as long as no human activities would open opportunities to regularly transport diaspores into the forest. However, in the land-use systems, the species has gained a wide distribution and abundance. The species is not regarded as an extremely noxious weed by farmers, and therefore, no special eradication measures are taken beside the general weed management in plantations [75]; pers. obs. of the author’s team. Hence, the species already has many potential source populations and a high propagule pressure exists in close vicinity of rainforest reservations. Although the invasive potential is at the moment regarded as moderate, the species may become rapidly invasive with human disturbance and continued deforestation of lowland rainforest systems in Sumatra.

## Supporting Information

S1 Fig Genetic diversity in land-use systems. Box plot summary of the genetic variation within the three land-use systems expressed as mean gene diversities per plot inferred from (a) Nei’s gene diversity (H_j_) and (b) panmictic heterozygosity (H_s_). Sample size (N) indicates number of plots which were analysed within each land-use system. Kruskal-Wallis test: (a) H = 4.263, *P* = 0.119; (b) H = 9.491, *P* = 0.009, Mann-Whitney comparisons: rubber x jungle rubber (*P* = 0.010/Bonferroni corrected *P* = 0.031), rubber x oil palm (*P* = 0.017/Bonferroni corrected *P* = 0.051).
(EPS)

S2 Fig Band richness within unequal samples. An estimate of the allelic richness (expressed as band richness after rarefaction to 5), was plotted against sample size. The both variables are not significantly correlated (*r* = -0.327, *P* = 0.172).
(EPS)

S1 Table List of populations. (XLSX)

S2 Table Correlation among population genetic variables. (XLSX)

S3 Table List of samples analyzed by flow cytometry. (XLSX)

S4 Table Flow cytometry analysis of leaves. (XLSX)

S5 Table Flow cytometry analysis of seeds. (XLSX)
